# Liu Shen Wan regulates the SPHK1/S1P axis to ameliorate influenza-induced inflammation via integrated network pharmacology and lipidomics

**DOI:** 10.3389/fimmu.2025.1764754

**Published:** 2026-01-16

**Authors:** Biao Lei, Zhenyang Liu, Peifang Xie, Xuanxuan Li, Zhanyu Cui, Ruihan Chen, Bin Liu, Shihua Chen, Yaxin Li, Min Liang, Hao Liang, Ai Li, Fanghao Zheng, Zifeng Yang, Qinhai Ma

**Affiliations:** 1 State Key Laboratory of Respiratory Disease, National Clinical Research Center for Respiratory Disease, Guangzhou Institute of Respiratory Health, The First Affiliated Hospital of Guangzhou Medical University, Guangzhou Medical University, Guangzhou, Guangdong, China; 2 Southern Medical University Hospital of Integrated Traditional Chinese and Western Medicine, Southern Medical University, Guangzhou, Guangdong, China; 3 The Eighth School of Clinical Medicine, Guangzhou University of Chinese Medicine, Foshan, Guangdong, China; 4 State Key Laboratory of Quality Research in Chinese Medicine, Macau University of Science and Technology, Taipa, Macao, Macao SAR, China; 5 Faculty of Innovation Engineering, Macau University of Science and Technology, Taipa, Macao, Macao SAR, China; 6 Department of Oncology, The Fifth Affiliated Hospital, Guangzhou Medical University, Guangzhou, Guangdong, China; 7 Baiyun District Maternal and Child Health Hospital, Guangzhou, Guangdong, China

**Keywords:** influenza virus, Liu shen wan, network pharmacology, sphingolipid metabolism, SPHK1/S1P axis

## Abstract

**Background:**

Liu Shen Wan (LSW) can modulate sphingolipid metabolism, which is a key pathway in inflammatory regulation, yet the precise mechanistic actions remain elusive. This study aimed to elucidate the mechanism by which LSW regulates sphingolipid metabolism to mitigate influenza-induced inflammatory responses.

**Methods:**

The potential mechanisms of LSW were initially predicted and validated via network pharmacology and lipidomics. A549 cells were infected with influenza A/Puerto Rico/8/34 (H1N1) (PR8) or transfected to overexpress sphingosine kinase-1 (SPHK1), then treated with LSW. *In vivo*, mice were infected with PR8 or challenged with rAAV9-SPHK1 and administered LSW for 5 days. Inflammatory factors and sphingolipid pathway-associated proteins were evaluated.

**Results:**

Network pharmacology identified sphingolipid signaling as a primary target of LSW. Lipidomics revealed LSW significantly reduced the levels of sphingomyelin (SM), ceramide, CerG2GNAc1, CerG3GNAc1, Ceramide phosphate and GM1 in lungs. In PR8-infected A549 cells, LSW significantly reduced sphingomyelinase (ASMase) and Ceramide (Cer) secretion. It also inhibited the expression of SPHK1 and sphingosine-1-phosphate (S1P) in A549 cells and in mice. Pharmacological inhibition of SPHK1 mirrored these anti-inflammatory effects. In SPHK1-overexpressing or TNF-α-stimulated A549 cells, LSW significantly attenuated the expression of SPHK1, CXCL10, and MCP-1. In the rAAV9-SPHK1 overexpression mouse model, LSW ameliorated lung pathological changes and reduced the expression of SPHK1, IFN-γ, and TNF-α.

**Conclusion:**

LSW alleviates influenza virus-induced inflammation by inhibiting the overactivation of the sphingolipid signaling pathway, specifically through targeting the SPHK1-S1P axis and ceramide-derived lipid mediators.

## 1 Introduction

Influenza remains a formidable global health challenge, caused by the influenza virus, and responsible for an estimated 3 to 5 million severe cases and 290 to 650 thousand deaths annually worldwide (1). Influenza A virus (IAV), in particular, is a major etiological agent capable of crossing species barriers and is frequently associated with severe clinical outcomes like pneumonia and acute respiratory distress syndrome (2). While vaccination serves as the primary strategy for prevention, its efficacy is often compromised by antigenic mismatch with circulating strains (3). Antiviral drugs, though available, face escalating challenges due to the evolution of drug-resistant influenza viruses (4). This underscores the urgent imperative to develop novel therapeutic strategies that can effectively mitigate influenza pathogenesis.

A hallmark of severe IAV infection is the onset of a dysregulated immune response characterized by a cytokine storm, which correlates strongly with disease severity and mortality (5). Emerging evidence indicates that IAV reprograms host metabolic pathways, including sphingolipid metabolism, to fuel excessive inflammation (6, 7). Sphingolipids, once regarded primarily as structural membrane components, are now recognized as dynamic signaling molecules regulating viral infection and inflammatory processes. Key plays include sphingomyelin (SM), ceramide (Cer), sphingosine (Sph) and sphingosine-1-phosphate (S1P). The enzymes acid sphingomyelinase (ASMase) and sphingosine kinase-1 (SPHK1) catalyze the formation of Cer and S1P, respectively, which in turn activate the NF-κB, MAPK, and PI3K/AKT signaling cascade (8–11). Notably, SPHK1 has become an important target against respiratory virus infection (8, 12), and its inhibition has been shown to protect mice from IAV infection (13). Despite these insights, no sphingolipid-targeting therapies have yet transitioned to clinical use, highlighting a critical gap in current treatment options.

Traditional Chinese medicines (TCMs) have been widely used to treat respiratory diseases in China (14–16). Reflecting the TCM principle of “clearing heat and detoxifying,” many formulations target inflammatory excess and immune dysregulation (17, 18). Liu Shen Wan (LSW), a classical prescription comprising Bezoar (the gall-stone of *Bos taurus domesticus Gmelin*), Musk (the excretion of *Moschus*), cinobufagin venom toad (the excretion of *Venenum Bufonis*), pearl (the shell of *Pernulo*), *realgar*, and borneol, is officially recommended in China for influenza treatment due to its potent heat-clearing and detoxifying properties (19). Multiple studies also indicate that LSW can suppress overactivated inflammatory response induced by IAV or *Staphylococcus aureus* (18, 20, 21). A recent clinical study further associated LSW’s anti-inflammatory effects with modulation of sphingolipid signaling (22). However, the precise mechanistic basis, especially its impact on sphingolipid metabolism remains unexplored.

Given the multi-component nature of TCM formulations, systems-level approaches are essential to decipher their mechanisms (23). Network pharmacology offers a powerful framework for identifying potential drug-disease interactions and mapping complex mechanisms of action (24, 25). Furthermore, lipidomics has emerged as a pivotal tool for elucidating how TCMs modulate lipid metabolic networks, including sphingolipids, in the context of infectious diseases (26). Integrating these methods can bridge traditional knowledge with contemporary molecular science, offering holistic insights into formula efficacy.

In this study, we employed an integrated strategy combining network pharmacology and lipidomics to investigate whether LSW ameliorates IAV-induced inflammation via regulation of the sphingolipid signaling pathway. We specifically focused on the SPHK1/S1P axis, which was a key nexus of inflammatory signaling, using *in vitro* and *in vivo* models of IAV infection. Our findings reveal that LSW significantly attenuates dysregulated sphingolipid metabolism and suppresses inflammatory responses, underscoring its potential as a multi-target agent against influenza-associated inflammation.

## 2 Materials and methods

### 2.1 Collection of components and targets

The active components and corresponding targets of LSW were identified in TCMSP databases as previously mentioned (24). All the compounds were screened using the criteria of Drug Likeness (DL) ≥ 0.18 and Oral Bioavailability (OB) ≥ 30%. In addition, the active components and targets were supplied in PubMed and HERB database.

Influenza-related targets were collected from Genecard, Drugbank, DisGeNET and CTD databases. The target name was corrected by the UniProt database. The potential targets of LSW against influenza were obtained via the online Venny 2.1 website (24).

### 2.2 Kyoto encyclopedia of genes and genomes pathway enrichment analysis

Potential therapeutic targets of LSW for influenza were analyzed in the DAVID database with the parameters set to Homo sapiens species and a significance threshold of *P* ≤ 0.05.

### 2.3 Molecular docking

To explore the key ingredients that exert pharmacological effects, the binding sites and binding activity of ingredients with SPHK1 were determined by the CB-Dock website. The compounds were selected according to the degree analyzed by Cytoscape software. SPHK1 (PDB format) was obtained from the PDB database and three-dimensional (3D) structures of key ingredients were acquired from the PubChem database. The vina scores and cavity size were used to evaluate the binding activity of ingredients with key targets, which could be obtained on the CB-Dock website. PyMOL software was used to analyze the molecular binding sites.

### 2.4 Reagents

LSW (lot: SA01004C) was offered by Suzhou Leiyunshang Medicine Pharmaceutical Co., Ltd. (Suzhou, China) and prepared as previously mentioned (27). In the previous study, the index components in LSW were detected by high-performance liquid chromatography (28). SPHK1 (lot: Ab262679) was purchased from Abcam. GAPDH (lot: 5174S) and β-Actin (lot: 3700) were purchased from CST. PF-543 hydrochloride (lot: S7177), a SPHK1 inhibitor, was purchased from Selleck.

### 2.5 Viruses and cells

A549 cells were purchased from American Type Culture Collection (ATCC, USA). A549 cells were cultured in DMEM/F12 (1:1) medium (Gibco, USA) with 10% fetal bovine serum. A549 cells were exposed to PR8 at a MOI of 1 for 60 min at 37 °C. After removing the inoculum, A549 cells were washed with PBS, then replaced with DMEM/F12 containing LSW(H) (1.5 μg/mL), LSW(M) (0.75 μg/mL), LSW(L) (0.375 μg/mL) or oseltamivir (OSE). During this process, A549 cells were infected with PR8 for 24 h to activate the sphingolipid signaling pathway. In addition, recombinant human TNF-α (Peprotech, lot:021825) was also used to stimulate A549 cells to activate the sphingolipid signaling pathway. A549 cells were stimulated with TNF-α (50 ng/mL) and treated with different doses of LSW for 24 h to detect the sphingolipid signaling pathway and cytokine expression.

### 2.6 Cell transfection

SPHK1 overexpression was achieved by transfecting A549 cells with plasmids containing the full-length human SPHK1 cDNA, which was designed and synthesized by AmyJet Scientific Inc. A549 cells (1.7 × 10^5^) were seeded into 12-well plates in an incubator for 24 h, followed by further transfection with the plasmids-transfection reagent mixture using Lipofectamine 3000™ (Thermo Fisher Scientific, Pittsburgh, PA) for 6 h. After that, the media were refreshed with DMEM/F12 containing 2% FBS. After transfection for 16 h, A549 cells were incubated with LSW or PF-543 hydrochloride for 24 h. The sequence of SPHK1 can be obtained in **Supplementary Table 1**.

### 2.7 Animal experiment

Specific pathogen-free female BALB/c mice (Certificate No. GZL0008) were purchased from Guangdong medical laboratory animal center (Guangdong, China). Mice weighing 18–20 g and aged 6 to 8 weeks were used for animal experiments. They were housed in collective cages at 22 ± 1°C with a relative humidity of 50 ± 10% and a 12-h light/dark cycle. Mice were randomly allocated into 6 groups including normal control (NC) group, PR8 group, OSE group, LSW(H) (100 mg/kg), LSW(M) (50 mg/kg) and LSW(L) (25 mg/kg). All the mice were anesthetized and inoculated intranasally with 50 µL of PR8 (1LD_50_) or PBS as previously described (24). After being infected with PR8 for 2 h, the infected mice were orally administered different doses of LSW, OSE or water daily for 5 days. All the mice were anesthetized and euthanized on day 6 after PR8 infection. The experiments involving in animals were carried out following the guidelines of the Ethics Committee of Guangzhou Medical University for the management of experimental animals and were approved by the Ethics Committee of Guangzhou Medical University (20230234).

### 2.8 Delivery of recombinant adeno-associated virus

The rAAV9 expressing 3Flag (rAAV9-3Flag) and SPHK1 (rAAV9- SPHK1) were provided by PackGene Technology (Guangzhou, China). Mice were randomly allocated into 3 groups including rAAV9-3Flag, rAAV9-SPHK1 and LSW(H) treatment group. Mice were intranasally challenged with the rAAV9-3Flag or rAAV9-SPHK1 (50 μL; 2 × 10¹¹ GC/mL). After 15 days of infection, mice were administered LSW(H) or PBS via gavage for 5 days. The body weight was monitored daily for 6 consecutive days. Lung tissues were obtained to analyze the mRNA expression of SPHK1 and inflammatory mediators. In addition, the histological changes were analyzed by hematoxylin and eosin (H&E) reagents.

### 2.9 Reverse transcription and quantitative real-time PCR

The total RNA from cell samples and the lung tissues were extracted by TRIzol reagent. Complementary DNA was synthesized from RNA samples by reverse transcription, followed by amplification using the SYBR Premix Ex Taq kit (Vazyme, Nanjing, China). The sequences of primers were shown in **Table 1**.

**Table 1:** Primers used for qRT-PCR primer sequence .

Target gene	Forward (5′-3′)	Reverse (5′-3′)
GAPDH	GAAGGTGAAGGTCGGAGTC	GAAGATGGTGATGGGATTTC
ASMase	CTGACTCTCGGGTTCTCTGG	AGGTTGATGGCGGTGAATAG
SPHK1	GCTGGCAGCTTCCTTGAACCAT	GTGTGCAGAGACAGCAGGTTCA
IL-6	ACTCACCTCTTCAGAACGAATTG	CCATCTTTGGAAGGTTCAGGTTG
IP-10	GAAATTATTCCTGCAAGCCAATTT	TCACCCTTCTTTTTCAT-TGTAGCA
MCP-1	CAAGCAGAAGTGGGTTCAGGAT	AGTGAGTGTTCAAGTCTTCGGAGTT
TNF-α	AACATCCAACCTTCCCAAACG	GACCCTAAGCCCCCAATTCTC
CCL5	TTGCCTGTTTCTGCTT	TGTAACTGCTGCTGTG

### 2.10 Immunofluorescence assay

A549 cells were exposed to PR8 for 60 min at 37°C. After that, the inoculum was removed and replaced with DMEM/F12 containing LSW or OSE. After incubation for 24 h, A549 cells were washed with PBS and fixed with 4% paraformaldehyde for 25 min. Subsequently, A549 cells were permeabilized by 1% Triton for 20 min, which were then blocked with 5% BSA for 40 min. After that, A549 cells were stained with anti-SPHK1 antibody overnight at 4°C, followed by incubation with secondary antibodies (lot: SA00003-2, Proteintech, USA). The nuclei were stained with proLong antifade mountant and reagents containing DAPI (Thermo Fisher Scientific, USA) for 10 min. The images were acquired using a microscope (Nikon, Japan).

### 2.11 Enzyme-linked immunosorbent assay

A549 cells were infected with PR8 and treated with LSW for 24 h. After that, the culture medium was collected and centrifuged at 11,000 rpm at 4°C for 6 min to obtain the supernatant. Subsequently, the concentrations of Cer were detected using ELISA kits (Shanghai, mlbio). Experiments were conducted following the manufacturer’s instructions. The lung tissues were homogenized and then were centrifuged at 11,000 rpm at 4°C for 15 min. The supernatants were obtained for detecting S1P (JONLNBIO, China). Experiments were conducted under the manufacturer’s instructions.

### 2.12 Western blot

Samples were lysed on ice with RIPA lysis buffer (lot: P0013B, Beyotime, Shanghai, China). Protein concentrations were measured using BCA protein assay kits (Thermo Fisher Scientific, USA). Western blots were conducted as previously mentioned (27). The primary antibodies, including SPHK1, GAPDH or β-Actin, were incubated overnight at 4°C, followed by incubation with the secondary antibodies for 1 h.

### 2.13 Immunohistochemistry

After being deparaffinized with xylene and rehydrated with gradient ethanol, lung tissue sections were soaked in 3% H_2_O_2_ for 10 min. Subsequently, the lung sections were incubated with 5% BSA at room temperature for 60 min. After that, the lung sections were stained with SPHK1 antibody for 16 h. After being washed with PBST, the lung sections were incubated with the corresponding secondary antibody at room temperature for 60 min. The immunoreactivity of SPHK1 protein was visualized by a DAB substrate kit and stained with hematoxylin. Finally, the images were obtained by inverted fluorescence microscopy (Leica, Germany).

### 2.14 Lipidomic analysis and data processing

Each sample was spiked with 20 µL of an internal standard mixture containing representative lipid classes (SPLASH^®^ LIPIDOMIX MASS SPRCSTANDARD, AVANTI, 330707-1EA), followed by the addition of 200 µL of water. The mixture was then vortexed for 10 s. Subsequently, 240 µL of precooled methanol was introduced, and the mixture was vortexed for an additional 30 s. Following this, 800 µL of pre-chilled methyl tert-butyl ether was added. Lipid extraction was performed by sonicating the mixture in an ice-cooled sonication bath for 20 min. The resulting mixture was then incubated at room temperature for 30 min and subsequently subjected to centrifugation at 14,000 g for 15 min at 10°C, after which the upper organic solvent layer was collected and evaporated to dryness under a nitrogen stream.

Reverse-phase chromatography was employed for liquid chromatography (LC) separation using a CSH C18 column (1.7 µm, 2.1 mm × 100 mm, Waters). Prior to analysis, the lipid extracts were dried under a gentle nitrogen stream and reconstituted in 200 µL of 90% isopropanol in acetonitrile. After centrifugation at 14,000 g for 20 min at 4°C, the clear supernatant was transferred to an LC-MS vial. A 3 µL aliquot was injected for analysis. The chromatographic separation employed a binary gradient with the following mobile phases: Solvent A, composed of acetonitrile and water (60:40, v/v) containing 0.1% formic acid and 0.1 mM ammonium formate; and Solvent B, composed of acetonitrile and isopropanol (10:90, v/v) containing 0.1% formic acid and 0.1 mM ammonium formate. The initial mobile phase was set at 40% solvent B with a flow rate of 300 μL/min for 3.5 min, which was then linearly increased to 75% solvent B over 9.5 min and further increased to 99% solvent B over 6 min, followed by re-equilibration at 40% solvent B for 5 min. Mass spectra were acquired using the Q-Exactive Plus instrument in both positive and negative ionization modes. The electrospray ionization parameters were optimized and standardized for all measurements as follows: the source temperature was maintained at 300°C, the capillary temperature was set at 350°C, the ion spray voltage was set to 3000 V, the S-Lens RF level was adjusted to 50%, and the scan range of the instrument was configured to m/z 200 -1800.

The raw LC-MS data were annotated using LipidSearch software. Principal component analysis (PCA), partial least squares-discriminant analysis (PLS-DA), and orthogonal partial least squares-discriminant analysis (OPLS-DA), were conducted using SIMCA software (Umetrics, Sweden). Differential metabolites were identified by combining the Variable Importance in Projection (VIP) value with the *P*-value (VIP > 1.0 and *P* < 0.05).

### 2.15 Statistical analysis

Statistical analysis was analyzed using GraphPad Prism 8. All quantitative data are reported as mean ± SD. To compare differences among groups, one-way ANOVA (Bonferroni or Dunnett’s test) was applied depending on the outcome of homogeneity of variance testing. The statistical significance was defined as *P* < 0.05.

## 3 Results

### 3.1 LSW could regulate sphingolipid signaling pathway during IAV infection based on network pharmacology

**Figure 1:**
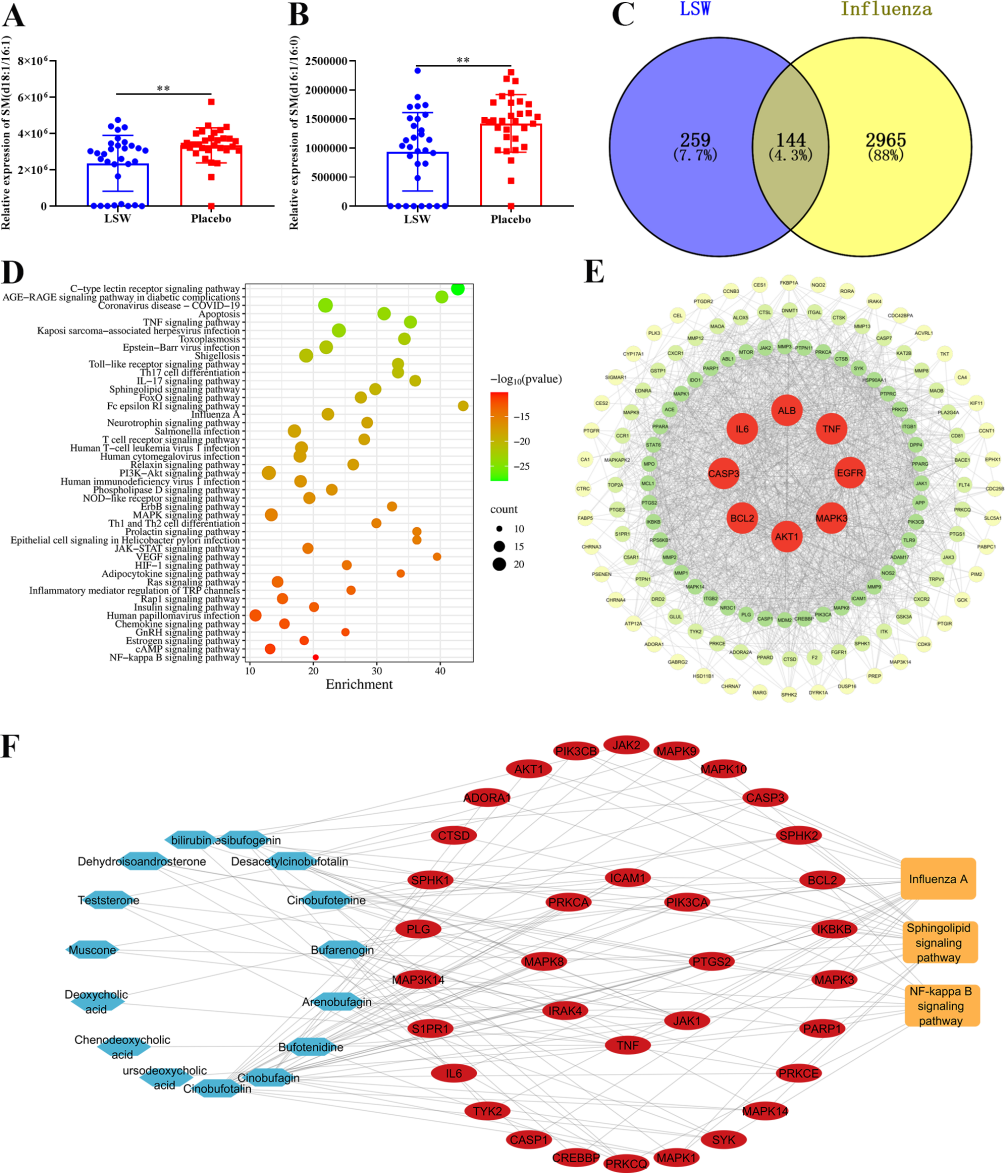
The potential mechanism of LSW in the treatment of influenza virus infection. **(A, B)** The relative expression of SM (d18:1/16:1) and SM (d18:1/16:0) in sera of flu patients. **(C)** The venn diagram of targets between 403 LSW-related targets and 3110 targets of influenza virus infection. **(D)** KEGG signaling enrichment analysis. **(E)** The protein–protein interactions of 144 targets. **(F)** The network of key active compounds, NF-κB signaling pathway, sphingolipid signaling pathway and influenza A signaling pathway. The data were shown as mean ± SD and analyzed by one-way ANOVA Bonferroni or Dunnett’s multiple comparisons tests (n=3). **, *p* < 0.01. *vs*. Placebo group.

LSW has previously been shown to suppress influenza virus-induced inflammatory response via inhibiting TLR4/NF-κB signaling pathway. Building on clinical evidence that LSW modulates sphingolipid metabolism in flu patients, particularly by reducing serum levels of SM (d18:1/16:1) and SM (d18:1/16:0) (**Figures 1A, B**), we applied network pharmacology to systematically elucidate its mechanism. The results showed that 144 potential influenza-related targets were regulated by LSW (**Figure 1C**). KEGG enrichment revealed significant involvement of key pathways including TNF, Toll-like receptor, NF-κB, and notably, the sphingolipid signaling pathway (**Figure 1D**). Among these targets, IL-6, TNF, and SPHK1, emerged as central regulators (**Figure 1E**). Further construction of a signaling pathway–target–compound network using Cytoscape highlighted SPHK1 as a critical node within the sphingolipid signaling pathway (**Figure 1F**), suggesting its pivotal role in mediating the anti-inflammatory effects of LSW.

### 3.2 LSW modulated PR8-induced dysregulated lipid metabolism in lungs

**Figure 2:**
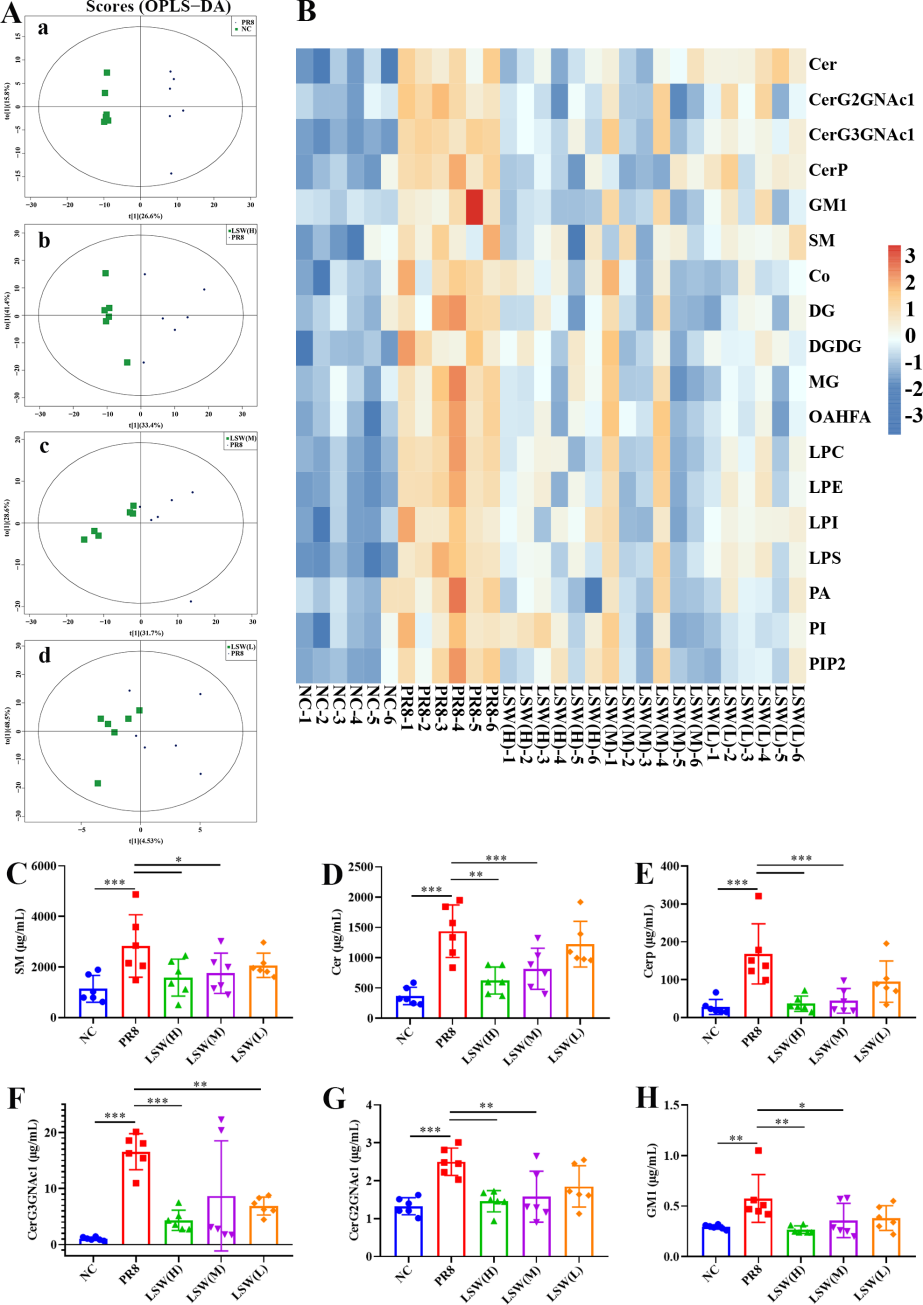
LSW alleviated lipid metabolism disorders in lungs during PR8 infection. **(A)** OPLS-DA score plot; **(B)** Clustering heatmap of the significantly differential metabolites; **(C-H)** The concentration of SM, Cer, Cerp, CerG3GNAc1, CerG2GNAc1 and GM1 in each group. The data were shown as mean ± SD and analyzed by one-way ANOVA Bonferroni or Dunnett’s multiple comparisons tests (n=3). *, *p* < 0.05; **, *p* < 0.01 or ***, *p* < 0.001. *vs*. PR8 group.

Network pharmacology analysis suggested that the sphingolipid signaling pathway may represent a key mechanism through which LSW exerts its anti-influenza effects. As the primary site of infection, the lung is likely where LSW ameliorates metabolic dysregulation, which may in turn modulate systemic sphingolipid imbalances observed in serum (**Figures 1A, B**). To test this, we employed quantitative lipidomics to assess whether LSW inhibits sphingolipid metabolism in the lungs. OPLS-DA revealed clear separation among the PR8-infected, LSW-treated, and normal control groups (**Figure 2A**), indicating significant modulation of lung lipid profiles by LSW. We identified seven major lipid categories in mouse lungs, including glycerophospholipids, sphingolipids, glycerolipids, sterol lipids, prenol lipids, fatty acyls and saccharolipids (**Supplementary Table 2**). Notably, LSW significantly reduced the levels of 18 lipid species (**Figure 2B**). Among these, six sphingolipids were markedly downregulated: SM, Cer, CerG2GNAc1, CerG3GNAc1, CerP, and GM1 (**Figures 2C-H**). The inhibitory effects of LSW on SM and Cer were similar to the effects in sera of flu patients (**Figures 1A, B**), reinforcing the translational relevance of our model. In addition, LSW suppressed a range of other dysregulated lipids involved in inflammatory and signaling pathways, including diglyceride (DG), monoglyceride (MG), etc. (**Supplementary Figure S1**). These results demonstrate that LSW comprehensively attenuates influenza-induced lipid metabolic disruption, with pronounced effects on sphingolipid pathways.

### 3.3 LSW could inhibit sphingolipid signaling pathway during PR8 infection

**Figure 3:**
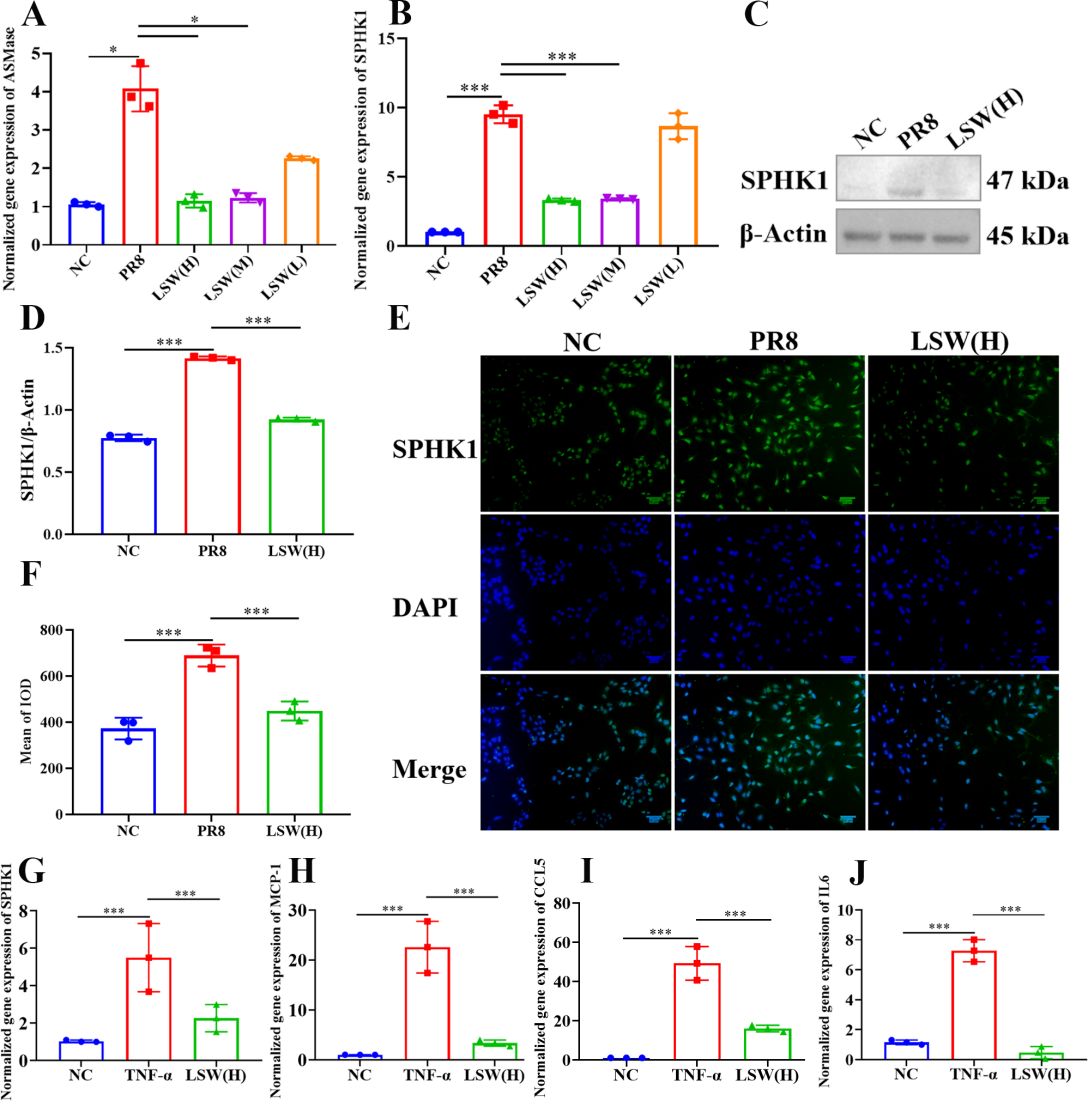
LSW inhibited sphingolipid metabolism *in vitro*. **(A)** The mRNA expression of ASMase in A549 cells during PR8 infection. **(B)** The mRNA expression of SPHK1 in A549 cells during PR8 infection. **(C)** The protein expression of SPHK1 in A549 cells during PR8 infection. **(D) **The relative expression of SPHK1 analyzed by Image J. **(E)** The protein expression of SPHK1 in A549 cells during PR8 infection; Scale bar = 50 μm. **(F)** The relative expression of SPHK1 analyzed by Image-Pro Plus 6.0. **(G)** The mRNA expression of SPHK1 in A549 cells stimulated by TNF-α. **(H)** The mRNA expression of MCP-1 in A549 cells stimulated by TNF-α. **(I)** The mRNA expression of CXCL10 in A549 cells stimulated by TNF-α. **(J)** The mRNA expression of IL6 in A549 cells stimulated by TNF-α. The data were shown as mean ± SD and analyzed by one-way ANOVA Bonferroni or Dunnett’s multiple comparisons tests (n=3). *, *p* < 0.05 or ***, *p* < 0.001. *vs*. PR8 group.

SPHK1 and ASMase play pivotal roles in the sphingolipid signaling pathway, where ASMase catalyzes the hydrolysis of SM to Cer, and SPHK1 converts sphingosine to S1P, forming a key signaling axis downstream of Cer. PR8 infection markedly activated this pathway, upregulating mRNA levels of both ASMase and SPHK1. Treatment with LSW significantly counteracted this effect, reducing their expression (**Figures 3A, B**). Consistent with ASMase inhibition, LSW also suppressed Cer secretion induced by PR8 infection (**Supplementary Figure S2**). Furthermore, western blot analysis confirmed that LSW downregulated SPHK1 protein expression in PR8-infected cells (**Figures 3C-E**).

Given that influenza infection triggers TNF-α secretion and that SPHK1 activation is essential for TNF-α–induced production of inflammatory mediators such as IL-6, MCP-1, and CCL5 (29), we evaluated whether LSW modulates this link. Notably, network pharmacology had previously identified the TNF signaling pathway as a target of LSW (**Figure 1**). Experimental results corroborated this prediction: LSW treatment not only decreased SPHK1 mRNA but also concurrently reduced the expression of MCP-1, CCL5 and IL6 (**Figures 3F-I**).

**Figure 4:**
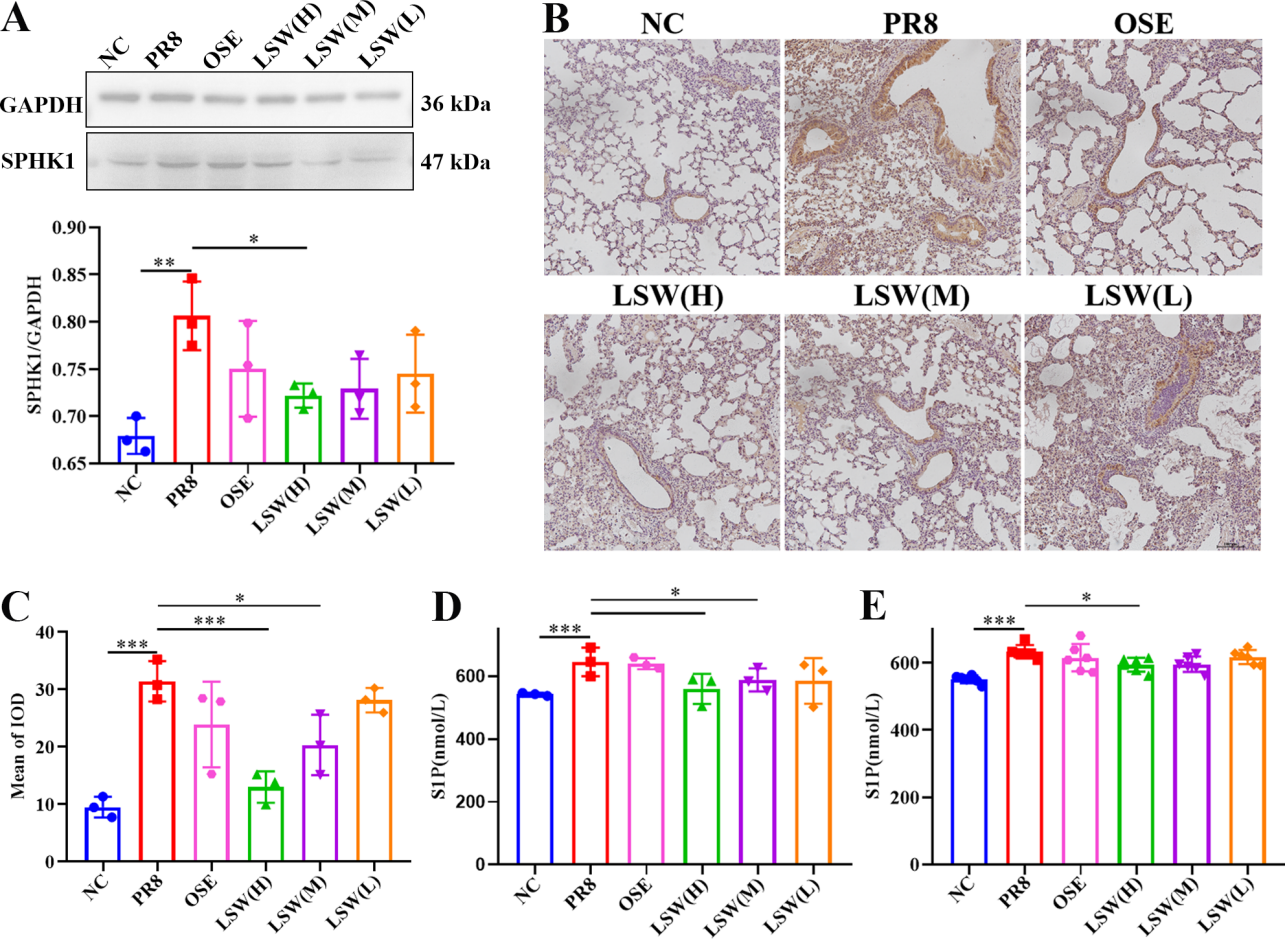
LSW inhibited SPHK1/S1P axis *in vivo* during PR8 infection. **(A)** The protein expression of SPHK1 in lungs during PR8 infection. **(B)** The protein expression and localization of SPHK1 in lungs during PR8 infection; Scale bar = 100 μm. **(C)** The relative expression of SPHK1 analyzed by Image-Pro Plus 6.0. **(D)** The production of S1P in lungs during PR8 infection. **(E)** The production of S1P in sera during PR8 infection. The data were shown as mean ± SD and analyzed by one-way ANOVA Bonferroni or Dunnett’s multiple comparisons tests (n=3). *, *p* < 0.05; **, *p* < 0.01 or ***, *p* < 0.001. *vs*. PR8 group.

Similar to the results of *in vitro* above, the protein expression of SPHK1 also upregulated in mouse lungs during PR8 infection, which could also be inhibited by LSW (**Figures 4A-C**). LSW could reduce the secretion of S1P upregulated in lungs or sera during PR8 infection (**Figures 4D-E**). These results indicated that LSW attenuates the inflammatory cascade by targeting SPHK1-dependent signaling.

### 3.4 LSW inhibited the overactivated inflammatory response by inhibiting SPHK1 expression during PR8 infection

**Figure 5:**
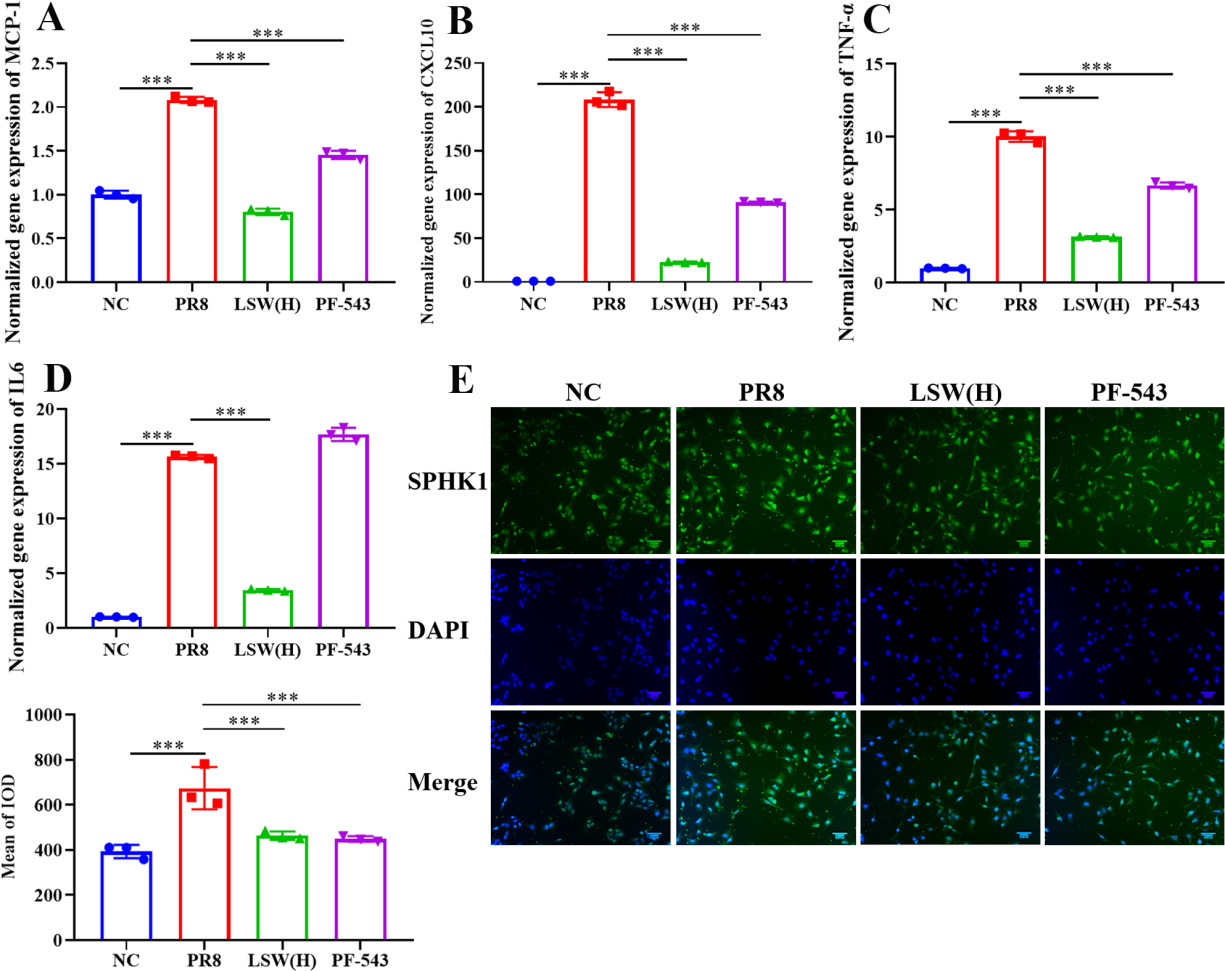
Inhibition of SPHK1 reduced the expression of cytokines and chemokines during PR8 infection. **(A)** The mRNA expression of MCP-1 in A549 cells during PR8 infection. **(B)** The mRNA expression of CXCL10 in A549 cells during PR8 infection. **(C)** The mRNA expression of TNF-α in A549 cells during PR8 infection. **(D)** The mRNA expression of IL6 in A549 cells during PR8 infection. **(E)** The protein expression of SPHK1 in A549 cells during PR8 infection; Scale bar = 50 μm. **(F)** The relative expression of SPHK1 analyzed by Image-Pro Plus 6.0. The data were shown as mean ± SD and analyzed by one-way ANOVA Bonferroni or Dunnett’s multiple comparisons tests (n=3). ***, *p* < 0.001. *vs*. PR8 group.

To elucidate the unknown role of SPHK1 in PR8-induced inflammation, we employed the specific SPHK1 inhibitor PF-543 hydrochloride in our experimental model. As shown in **Figures 5A-D**, PF-543 treatment significantly suppressed the expression of SPHK1, CXCL10, MCP-1 and TNF-α. These results firmly establish SPHK1 as a critical regulator of IAV-induced inflammation and highlight its therapeutic potential as a pharmacological target. Consistent with transcriptional regulation, LSW(H) also reduced the corresponding protein levels induced by SPHK1 overexpression (**Figure 5E**).

### 3.5 LSW inhibited the inflammation induced by SPHK1 overexpression

**Figure 6:**
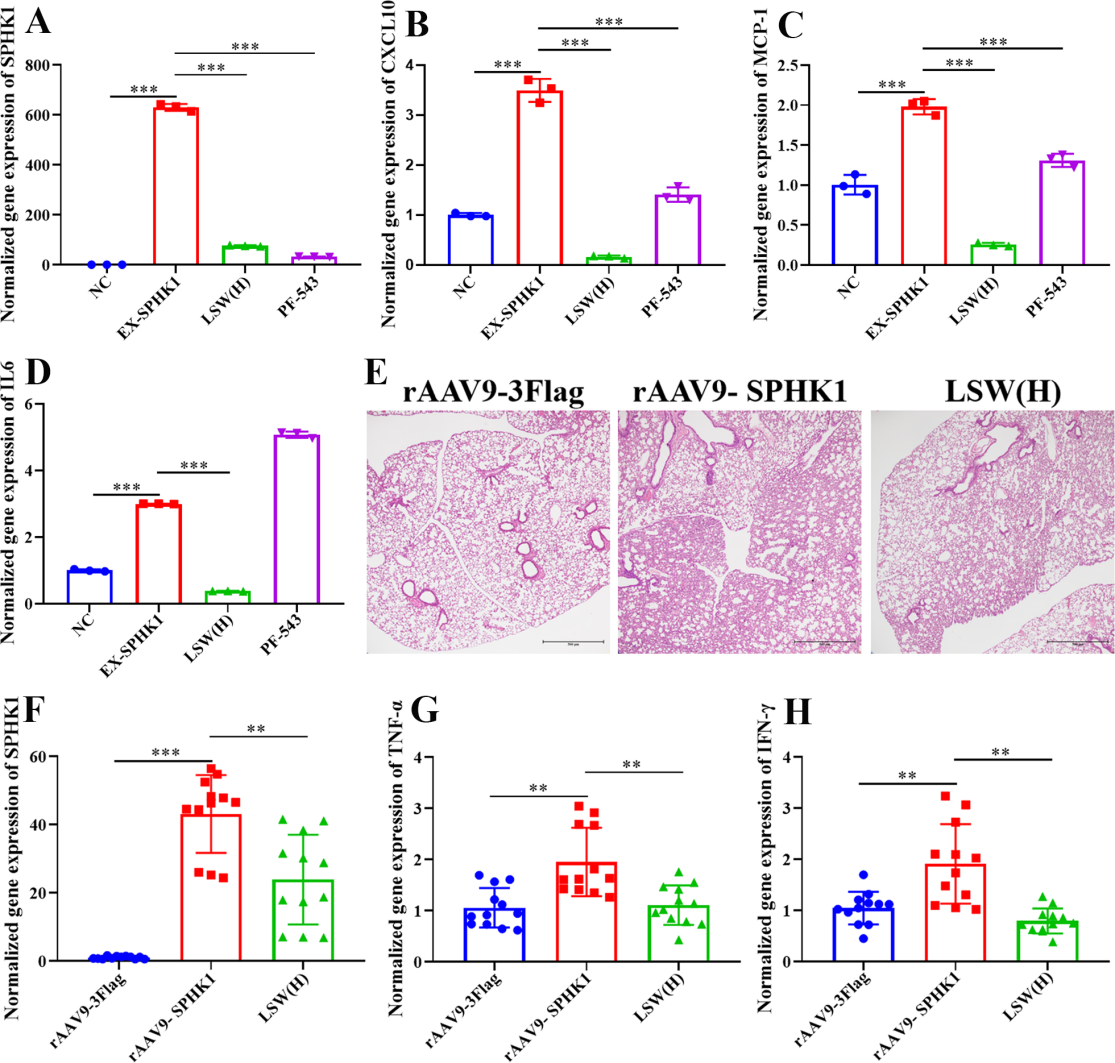
LSW inhibited the expression of SPHK1, cytokines and chemokines induced by SPHK1 overexpression. **(A)** The mRNA expression of SPHK1 induced by SPHK1 overexpression in A549 cells. **(B)** The mRNA expression of CXCL10 induced by SPHK1 overexpression in A549 cells. **(C)** The mRNA expression of MCP-1 induced by SPHK1 overexpression in A549 cells. **(D)** The mRNA expression of IL6 induced by SPHK1 overexpression in A549 cells. **(E)** H&E staining of lungs after delivery of rAAV; Scale bar = 500 μm. **(F)** The expression of SPHK1 in lungs after delivery of rAAV. **(G)** The expression of TNF-α in lungs after delivery of rAAV. **(H)** The expression of IFN-γ in lungs after delivery of rAAV. The data were shown as mean ± SD and analyzed by one-way ANOVA Bonferroni or Dunnett’s multiple comparisons tests (n=3). **, *p* < 0.01 or ***, *p* < 0.001. *vs*. PR8 group.

To further validate that LSW attenuates influenza-induced inflammation specifically through the sphingolipid signaling pathway, we overexpressed SPHK1 in A549 cells. SPHK1 overexpression markedly upregulated the expression of SPHK1, MCP-1, and IP-10, an effect that was significantly reversed by both high-dose LSW [LSW(H)] and the SPHK1 inhibitor PF-543 (**Figures 6A-D**).

These *in vitro* findings were corroborated *in vivo*. Mice administered rAAV9-SPHK1 exhibited substantially elevated pulmonary SPHK1 expression compared to the rAAV9-3Flag control group. Importantly, treatment with LSW(H) significantly suppressed the expression of SPHK1 and the levels of key inflammatory cytokines (IFN-γ and TNF-α) in the lungs of these animals (**Figures 6E-H**), underscoring the therapeutic potential of LSW in modulating SPHK1-driven inflammation.

### 3.6 The binding activities of key compounds with SPHK1 by molecular docking analysis

**Figure 7:**
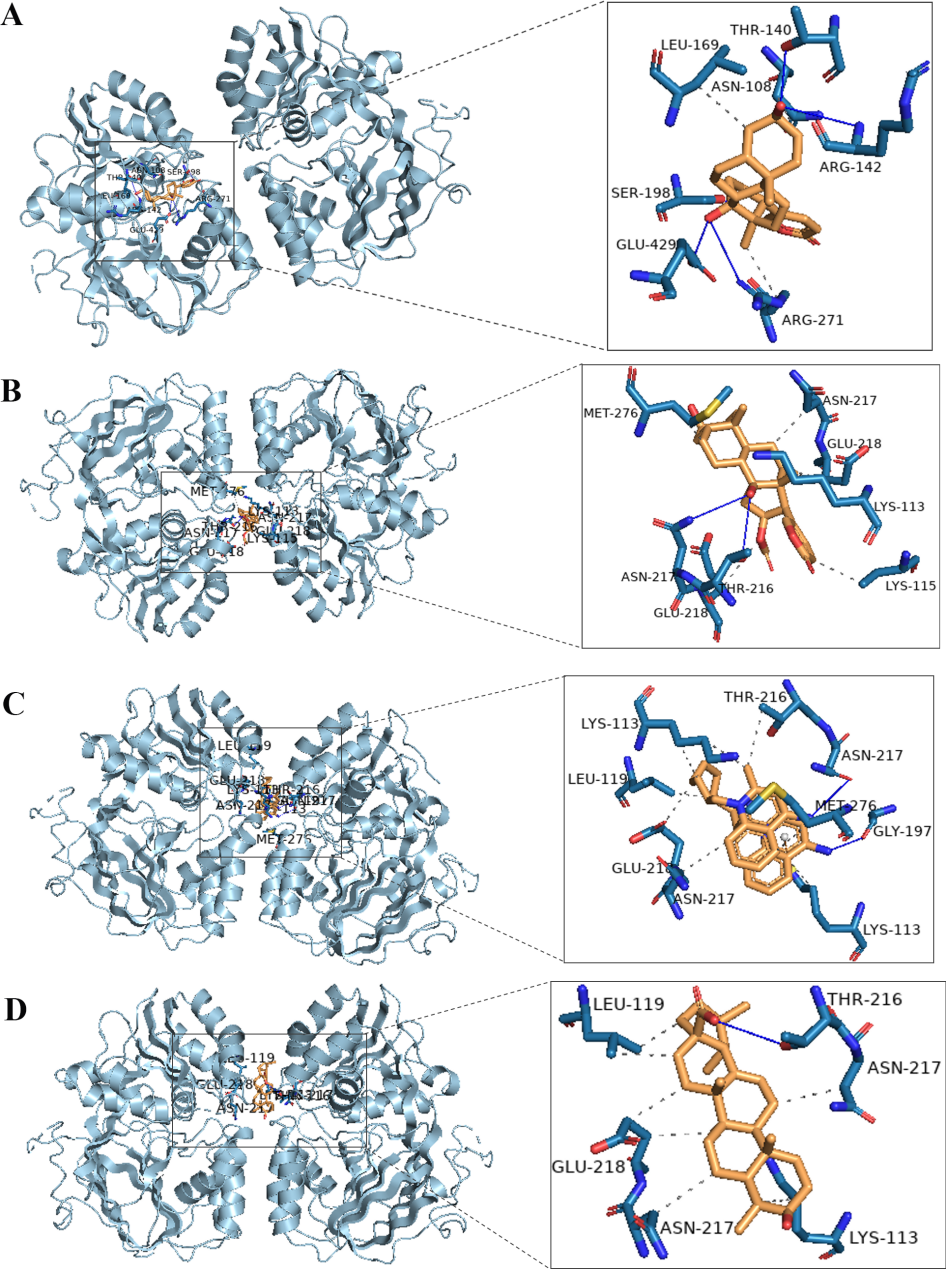
The interaction of the key compound with SPHK1. **(A)** The interaction of bufalin (golden) with SPHK1 (gray); **(B)** The interaction of bufotalin (golden) with SPHK1 (gray); **(C)** The interaction of decamine (golden) with SPHK1 (gray); **(D)** The interaction of ursolic acid (golden) with SPHK1 (gray).

To elucidate the molecular mechanism by which LSW modulates sphingolipid signaling, the molecular docking technique was utilized to analyze the binding activities of key compounds with SPHK1. Using the CB-Dock platform, binding affinity was assessed based on Vina scores (lower values indicating stronger stability) and cavity size (larger values reflecting higher accuracy). Multiple compounds exhibited robust binding potential to SPHK1 (**Table 2**), with bufalin, bufotalin decamine, and ursolic acid showing the highest binding affinities. The precise binding modes and molecular interactions of these compounds within the SPHK1 active site were depicted in **Figure 7**, suggesting their role as critical mediators of the anti-inflammatory effects of LSW through inhibition of SPHK1.

**Table 2:** The vina scores and cavities size of key compounds with SPHK1.

SPHK1 (PDB ID: 4v24)				
Chemical name	Degree	InChI Key	Vina scores	Cavity size
bufalin	89	QEEBRPGZBVVINN-BMPKRDENSA-N	-10	3813
androst-4-Ene-3,17-Dione	52	AEMFNILZOJDQLW-QAGGRKNESA-N	-8.9	3813
testosterone	45	MUMGGOZAMZWBJJ-DYKIIFRCSA-N	-9	3813
cinobufagin	39	SCULJPGYOQQXTK-OLRINKBESA-N	-8.8	3813
ursolic acid	38	WCGUUGGRBIKTOS-GPOJBZKASA-N	-9.2	3813
decamine	31	LTNZEXKYNRNOGT-UHFFFAOYSA-N	-9.4	3813
GABA	30	BTCSSZJGUNDROE-UHFFFAOYSA-N	-4.4	1633
chenodeoxycholic acid	28	RUDATBOHQWOJDD-BSWAIDMHSA-N	-9	3813
bufotalin	28	VOZHMAYHYHEWBW-NVOOAVKYSA-N	-9.9	3813
methoxybufotenin	27	ZSTKHSQDNIGFLM-UHFFFAOYSA-N	-6.9	1633

## 4 Discussion

Influenza virus is one of the most common virus strains causing pneumonia, high mortality and high cost (30, 31). The overactivated inflammatory responses are hallmark pathological features of influenza virus infection. Excessive inflammation can result in the formation of a “cytokine storm”, which can recruit a large number of inflammatory cells to sites of infection (2). These inflammatory cells such as neutrophils and macrophages, can destroy normal lung tissues by releasing ROS, matrix metalloproteinases, etc (32). Cytokine storm is one of the key factors contributing to high mortality in flu patients. Various studies have indicated that the sphingolipid signaling pathway is associated with the overactivated inflammatory response and involved in driving disease progression during respiratory viral infection (7, 8, 33, 34). Sphingolipid metabolites such as S1P, have served as key players in driving inflammatory signaling and immune responses. S1P can directly bind to TRAF2 to activate the NF-κB signaling pathway. In addition, S1P can be secreted extracellularly to activate the NF-κB signaling via the S1P-S1PR axis (35). As the pivotal regulator of S1P synthesis, SPHK1 is critically involved in overactivated inflammatory response during viral infection. Viral challenge activates SPHK1 in epithelial cells, perturbing S1P metabolic homeostasis. The release of S1P activates pro-inflammatory signaling pathways, which fuels a feedforward cycle of excessive inflammation (36). Suppression of SPHK1 can reduce the production of inflammatory mediators in A549 cells stimulated by TNF-α (29). IAV infection can increase the expression of SPHK1, accompanied by increased production of S1P, cytokines and chemokines. Specific SPHK1 inhibitor and overexpression of SPHK1 confirmed that inhibition of SPHK1 also reduces the expression of cytokines and chemokines in A549 cells during PR8 infection. Our results demonstrate that both pharmacological inhibition and genetic overexpression of SPHK1 modulate the expression of inflammatory mediators, underscoring the pivotal role of the SPHK1/S1P axis in influenza pathogenesis. It is important to note that LSW, as a multi-herb formulation, is likely to exert pleiotropic immunomodulatory effects beyond SPHK1 inhibition. The overall therapeutic benefit probably results from a synergistic modulation of multiple targets within the inflammatory network.

While antiviral drugs remain first-line treatments, they often fail to mitigate excessive inflammation in severe cases. LSW, a traditional Chinese formula renowned for its anti-inflammatory properties, has shown efficacy in reducing hyperinflammation induced by IAV, SARS-CoV-2, and *Staphylococcus aureus*, primarily via NF-κB inhibition (18, 20, 21, 27). Using integrated network pharmacology and lipidomics, we identified the sphingolipid signaling pathway as a key target of LSW. Specifically, SPHK1 emerged as a critical node targeted by LSW. We further validated that LSW downregulates SPHK1 expression and S1P production, leading to reduced levels of cytokines and chemokines in A549 cells and in mice. Molecular docking analyses revealed that bufalin, bufotalin decamine and ursolic acid act as potent inhibitors of SPHK1, implying direct roles in regulating sphingolipid metabolism.

Various types of bioactive lipid mediators are also important modulators of inflammatory response during influenza virus infection in addition to S1P (37, 38). Cer are sphingolipids that play a role in driving the pathological processes such as destroying the alveolar endothelial barrier. The accumulation of Cer can induce the pyroptosis of endothelial cells by activating the TXNIP/NLRP3/GSDMD signaling pathway accompanied by the release of inflammatory mediators (37, 39). Inhibition of the production of Cer may also contribute to the anti-inflammatory effects of LSW. Whether LSW directly or indirectly inhibits the activation of ASMase remains to be further elucidated. In addition, recent findings strongly suggest that numerous enveloped RNA viruses such as influenza virus, have adapted to utilize sphingolipids or glycosphingolipids for entry, replication or the creation of new virus particles (40). The modulation of sphingolipids or glycosphingolipids is a viable strategy to inhibit the replication of influenza virus. A previous study has found that LSW could inhibit the replication of influenza virus in MDCK cells and in mice (18). LSW could inhibit the production of sphingolipids (SM), glycosphingolipids (Cer, Cerp and GM1) and neutral glycosphingolipids (CerG3GNAc1 and CerG2GNAc1) in lungs during PR8 infection. The inhibitory effects of LSW on viral replication may be associated with suppression of the production of sphingolipids or glycosphingolipids.

Based on the correlative nature of our current findings, future studies will focus on establishing direct causality. To definitively prove that SPHK1 downregulation is a key mechanistic pathway through which LSW exerts its anti-inflammatory effects, we will employ both pharmacological and genetic rescue approaches. First, exogenous S1P supplementation experiments will be conducted to determine if it can reverse the anti-inflammatory outcomes of LSW treatment in infected cells and animal models. Second, utilizing SPHK1-knockout cells or mice will provide crucial genetic evidence, isolating the specific role of this enzyme. Together, these experiments will clarify whether the observed reduction in SPHK1 activity and subsequent S1P signaling is essential for the therapeutic action of LSW.

## 5 Conclusion

**Figure 8:**
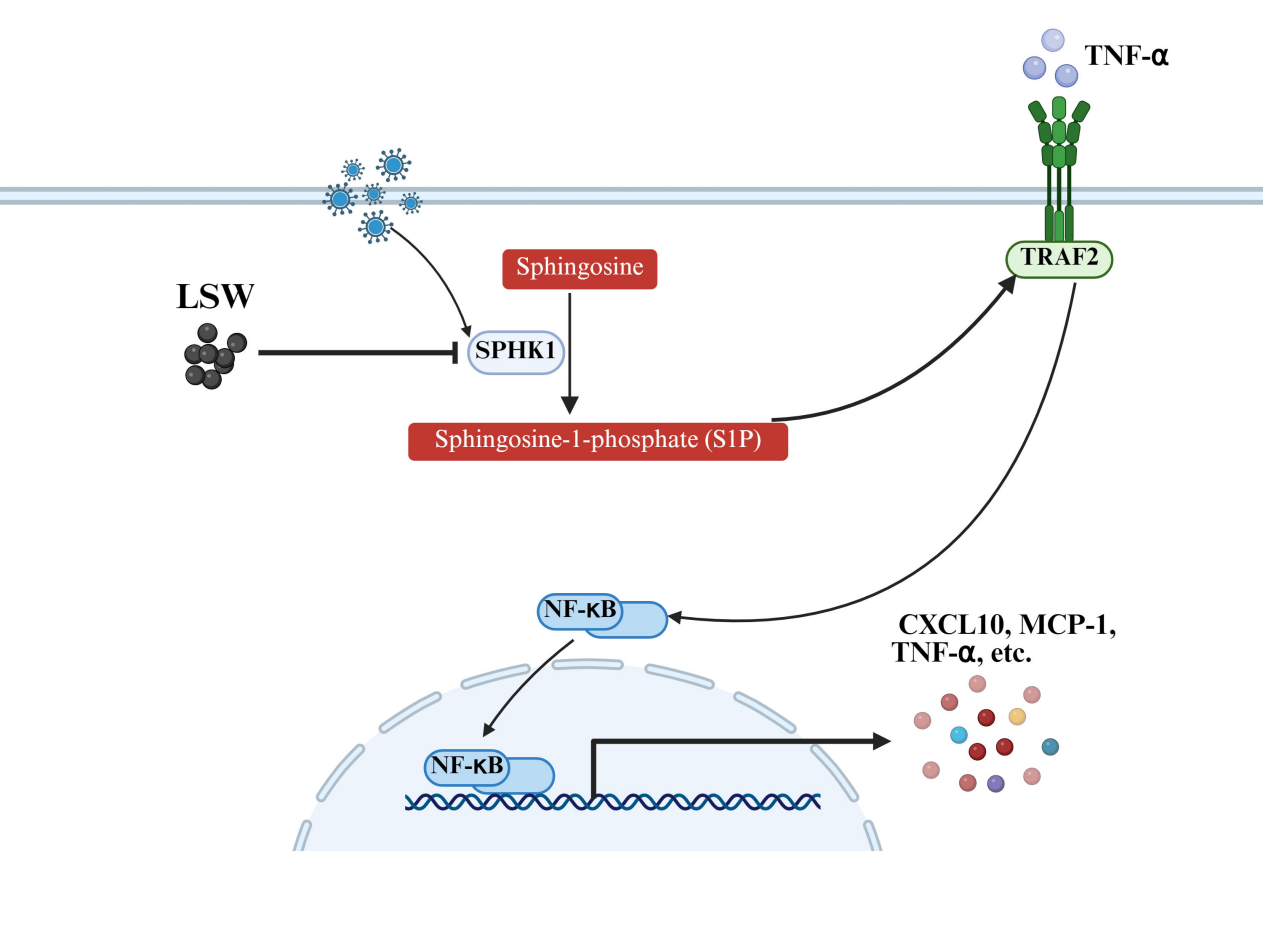
Schematic representation of the mechanisms of LSW on influenza virus infection. The sphingolipid signaling pathway was activated during influenza virus infection, accompanied with the release of cytokines and chemokines. LSW alleviated virus-induced overactivated inflammatory response by inhibiting SPHK1/S1P axis.

Our findings establish SPHK1 as a critical mediator of IAV-induced inflammation and demonstrate that LSW exerts potent anti-inflammatory effects by selectively inhibiting the SPHK1/S1P signaling axis (**Figure 8**). This study provides mechanistic evidence that LSW alleviates cytokine-driven lung pathology through modulation of sphingolipid metabolism. Furthermore, by integrating lipidomics with functional validation, we offer a systems-level perspective on how traditional medicine can regulate host responses to viral infection, highlighting the value of multi-omics approaches in ethnopharmacological research.

## Data Availability

The original contributions presented in the study are included in the article/**Supplementary Material**. Further inquiries can be directed to the corresponding authors.

## References

[1] WHO . WHO unveils new worldwide strategy against influenza (2019).

[2] Uyeki TM Hui DS Zambon M Wentworth DE Monto AS . Influenza. Lancet. (2022) 400:693–706. doi: 10.1016/S0140-6736(22)00982-5, PMID: 36030813 PMC9411419

[3] Uyeki TM Bernstein HH Bradley JS Englund JA File TM Fry AM . Clinical practice guidelines by the infectious diseases society of america: 2018 update on diagnosis, treatment, chemoprophylaxis, and institutional outbreak management of seasonal influenzaa. Clin Infect Dis. (2019) 68:e1–e47. doi: 10.1093/cid/ciy866, PMID: 30566567 PMC6653685

[4] Kumari R Sharma SD Kumar A Ende Z Mishina M Wang Y . Antiviral approaches against influenza virus. Clin Microbiol Rev. (2023) 36:e0004022. doi: 10.1128/cmr.00040-22, PMID: 36645300 PMC10035319

[5] Keshavarz M Solaymani-Mohammadi F Namdari H Arjeini Y Mousavi MJ Rezaei F . Metabolic host response and therapeutic approaches to influenza infection. Cell Mol Biol lett. (2020) 25:15. doi: 10.1186/s11658-020-00211-2, PMID: 32161622 PMC7059726

[6] Ning P Zheng Y Luo Q Liu X Kang Y Zhang Y . Metabolic profiles in community-acquired pneumonia: developing assessment tools for disease severity. Crit Care. (2018) 22:130. doi: 10.1186/s13054-018-2049-2, PMID: 29759075 PMC5952829

[7] Wendt CH Castro-Pearson S Proper J Pett S Griffin TJ Kan V . Metabolite profiles associated with disease progression in influenza infection. PloS One. (2021) 16:e0247493. doi: 10.1371/journal.pone.0247493, PMID: 33798209 PMC8018623

[8] Thomas S Samuel SV Hoch A Syphurs C Diray-Arce J . The implication of sphingolipids in viral infections. Int J Mol Sci. (2023) 24:17303. doi: 10.3390/ijms242417303, PMID: 38139132 PMC10743733

[9] Xia P Wadham C . Sphingosine 1-phosphate, a key mediator of the cytokine network: juxtacrine signaling. Cytokine Growth factor Rev. (2011) 22:45–53. doi: 10.1016/j.cytogfr.2010.09.004, PMID: 21051273

[10] Hammad SM Crellin HG Wu BX Melton J Anelli V Obeid LM . Dual and distinct roles for sphingosine kinase 1 and sphingosine 1 phosphate in the response to inflammatory stimuli in RAW macrophages. Prostaglandins other Lipid Mediators. (2008) 85:107–14. doi: 10.1016/j.prostaglandins.2007.11.002, PMID: 18166496 PMC2290737

[11] Schütze S Potthoff K Machleidt T Berkovic D Wiegmann K Krönke M . TNF activates NF-kappa B by phosphatidylcholine-specific phospholipase C-induced “acidic” sphingomyelin breakdown. Cell. (1992) 71:765–76. doi: 10.1016/0092-8674(92)90553-o, PMID: 1330325

[12] Khan RJ Single SL Simmons CS Athar M Liu Y Bodduluri S . Altered sphingolipid pathway in SARS-CoV-2 infected human lung tissue. Front Immunol. (2023) 14:1216278. doi: 10.3389/fimmu.2023.1216278, PMID: 37868972 PMC10585362

[13] Xia C Seo YJ Studstill CJ Vijayan M Wolf JJ Hahm B . Transient inhibition of sphingosine kinases confers protection to influenza A virus infected mice. Antiviral Res. (2018) 158:171–7. doi: 10.1016/j.antiviral.2018.08.010, PMID: 30125617 PMC6190705

[14] Zhao Qianru ZR Zihan G Lei B Shanshan G Yu W Xiaolan C . Xuanfei Baidu granule alleviates coronavirus-induced pneumonia in low-temperature and high-humidity environments. Acupunct Herbal Med. (2023) 3:200–6. doi: 10.1097/HM9.0000000000000068

[15] Chen Xiaopeng WM . Traditional Chinese medicine during the COVID-19 pandemic: Recent successes and future perspectives. Acupunct Herbal Med. (2023) 3:357–9. doi: 10.1097/HM9.0000000000000084

[16] Yang KJB Bautista Liana YABS Iben DGBS Tran DHBS . The role of ginseng as an anti-asthmatic agent. Acupunct Herbal Med. (2024) 4:358–66. doi: 10.1097/HM9.0000000000000123

[17] Lei B Mu J Xu G Yang X Huang W Hu L . Jing-Yin-Gu-Biao formula protects mice from postinfluenza Staphylococcus aureus infection by ameliorating acute lung injury and improving hypercoagulable state via inhibiting NETosis. Front Immunol (2025) 16:1567522. doi: 10.3389/fimmu.2025.1567522, PMID: 40134435 PMC11933027

[18] Ma Q Huang W Zhao J Yang Z . Liu Shen Wan inhibits influenza a virus and excessive virus-induced inflammatory response via suppression of TLR4/NF-kappaB signaling pathway *in vitro* and *in vivo*. J Ethnopharmacol. (2020) 252:112584. doi: 10.1016/j.jep.2020.112584, PMID: 31972325

[19] Branch CMAEM . ‘Liushengwan (capsules)’ Clinical application in acute infectious diseases: emergency expert consensus. Chin J Emergency Med. (2022) 31:5. doi: 10.3760/cma.j.issn.1671-0282.2022.02.006

[20] Zhao J Wang Y Huang X Ma Q Song J Wu X . Liu Shen Wan inhibits influenza virus-induced secondary *Staphylococcus aureus* infection *in vivo* and *in vitro*. J Ethnopharmacol. (2021) 277:114066. doi: 10.1016/j.jep.2021.114066, PMID: 33766755

[21] Song Y Ma Q Luo J Yang Z Li J Zhao J . Liushen Wan alleviates the virulence and inflammation of *Staphylococcus aureus* via NLRP3 inflammasome and TLR2-NF-κB/p38 MAPK signaling pathways. Int Immunopharmacol. (2025) 144:113633. doi: 10.1016/j.intimp.2024.113633, PMID: 39566390

[22] Ma Q Chen R Zeng J Lei B Ye F Wu Q . Investigating the effects of Liushen Capsules (LS) on the metabolome of seasonal influenza: A randomized clinical trial. Front Pharmacol. (2022) 13:968182. doi: 10.3389/fphar.2022.968182, PMID: 36034844 PMC9402892

[23] Zhang Junhua DE Liu L Tian J Chen S Zhu L Zhang B . Ten recommendations for the high-quality development of traditional Chinese medicine in the new era. Acupunct Herbal Med. (2025) 5:131–3. doi: 10.1097/HM9.0000000000000162

[24] Lei B Su Y Chen R Chen Z Liu B Chen Y . Uncovering the mechanisms of baBaoWuDanYaoMo against influenza A virus and virus-induced inflammation based on network pharmacology and pharmacological evaluation. Infect Drug Resist. (2025) 18:567–87. doi: 10.2147/IDR.S491101, PMID: 39902273 PMC11789520

[25] Zhang Junhua CX Luqi H Liu L Wang Q Tian J Zhu L . Wuzhen consensus on traditional chinese medicine and artificial intelligence. Acupunct Herbal Med. (2025) 5:134–5.

[26] Zheng Y Zhang Y Chen Y Deng X Liu B Xu Q . Indoleamine 2,3-dioxygenase 1 drives epithelial cells ferroptosis in influenza-induced acute lung injury. Redox Biol. (2025) 81:103572. doi: 10.1016/j.redox.2025.103572, PMID: 40023977 PMC11915170

[27] Ma Q Lei B Chen R Liu B Lu W Jiang H . Liushen Capsules, a promising clinical candidate for COVID-19, alleviates SARS-CoV-2-induced pulmonary *in vivo* and inhibits the proliferation of the variant virus strains *in vitro*. Chin Med. (2022) 17:40. doi: 10.1186/s13020-022-00598-4, PMID: 35365215 PMC8972667

[28] Li QLNC Dai JJ Di LQ Wang HB Yang YQ Li JS . A method for detecting the Liu Shen pill by HPLC. Chinese patent patent CN109765319A. (2019). 2019-05-17.

[29] Billich A Bornancin F Mechtcheriakova D Natt F Huesken D Baumruker T . Basal and induced sphingosine kinase 1 activity in A549 carcinoma cells: function in cell survival and IL-1beta and TNF-alpha induced production of inflammatory mediators. Cell Signaling. (2005) 17:1203–17. doi: 10.1016/j.cellsig.2004.12.005, PMID: 16038795

[30] Paget J Staadegaard L Wang X Li Y van Pomeren T van Summeren J . Global and national influenza-associated hospitalization rates: Estimates for 40 countries and administrative regions. J Global Health. (2023) 13:04003. doi: 10.7189/jogh.13.04003, PMID: 36701368 PMC9879557

[31] Li ZJ Zhang HY Ren LL Lu QB Ren X Zhang CH . Etiological and epidemiological features of acute respiratory infections in China. Nat Commun. (2021) 12:5026. doi: 10.1038/s41467-021-25120-6, PMID: 34408158 PMC8373954

[32] Malireddi RKS Sharma BR Kanneganti TD . Innate immunity in protection and pathogenesis during coronavirus infections and COVID-19. Annu Rev Immunol. (2024) 42:615–45. doi: 10.1146/annurev-immunol-083122-043545, PMID: 38941608 PMC11373870

[33] Uranbileg B Isago H Nakayama H Jubishi D Okamoto K Sakai E . Comprehensive metabolic modulations of sphingolipids are promising severity indicators in COVID-19. FASEB J: Off Publ Fed Am Soc Exp Biol. (2024) 38:e23827. doi: 10.1096/fj.202401099R, PMID: 39012295

[34] McGowan EM Haddadi N Nassif NT Lin Y . Targeting the sphK-S1P-SIPR pathway as a potential therapeutic approach for COVID-19. Int J Mol Sci. (2020) 21:7189. doi: 10.3390/ijms21197189, PMID: 33003377 PMC7583882

[35] Mohd A Atahar H Mohammad K Mohammed S Yusuf A Abdur RJDDT . Sphingosine-1-phosphate signaling: unraveling its role as a drug target against infectious diseases. Drug Discov Today. (2015) 21:133–42. doi: 10.1016/j.drudis.2015.09.013, PMID: 26456576

[36] Seo YJ Pritzl CJ Vijayan M Bomb K McClain ME Alexander S . Sphingosine kinase 1 serves as a pro-viral factor by regulating viral RNA synthesis and nuclear export of viral ribonucleoprotein complex upon influenza virus infection. PloS One. (2013) 8:e75005. doi: 10.1371/journal.pone.0075005, PMID: 24137500 PMC3796690

[37] Jiang J Shi Y Cao J Lu Y Sun G Yang J . Role of ASM/Cer/TXNIP signaling module in the NLRP3 inflammasome activation. Lipids Health dis. (2021) 20:19. doi: 10.1186/s12944-021-01446-4, PMID: 33612104 PMC7897379

[38] Tam VC Quehenberger O Oshansky CM Suen R Armando AM Treuting PM . Lipidomic profiling of influenza infection identifies mediators that induce and resolve inflammation. Cell. (2013) 154:213–27. doi: 10.1016/j.cell.2013.05.052, PMID: 23827684 PMC3753192

[39] Liu F Zhang Y Shi Y Xiong K Wang F Yang J . Ceramide induces pyroptosis through TXNIP/NLRP3/GSDMD pathway in HUVECs. BMC Mol Cell Biol. (2022) 23:54. doi: 10.1186/s12860-022-00459-w, PMID: 36517743 PMC9749313

[40] Yager EJ Konan KV . Sphingolipids as potential therapeutic targets against enveloped human RNA viruses. Viruses. (2019) 11:912. doi: 10.3390/v11100912, PMID: 31581580 PMC6832137

