# Correction: Liu Shen Wan regulates the SPHK1/S1P axis to ameliorate influenza-induced inflammation via integrated network pharmacology and lipidomics

**DOI:** 10.3389/fimmu.2026.1809234

**Published:** 2026-02-18

**Authors:** Biao Lei, Zhenyang Liu, Peifang Xie, Xuanxuan Li, Zhanyu Cui, Ruihan Chen, Bin Liu, Shihua Chen, Yaxin Li, Min Liang, Hao Liang, Ai Li, Fanghao Zheng, Zifeng Yang, Qinhai Ma

**Affiliations:** 1 State Key Laboratory of Respiratory Disease, National Clinical Research Center for Respiratory Disease, Guangzhou Institute of Respiratory Health, The First Affiliated Hospital of Guangzhou Medical University, Guangzhou Medical University, Guangzhou, Guangdong, China; 2 Southern Medical University Hospital of Integrated Traditional Chinese and Western Medicine, Southern Medical University, Guangzhou, Guangdong, China; 3 The Eighth School of Clinical Medicine, Guangzhou University of Chinese Medicine, Foshan, Guangdong, China; 4 State Key Laboratory of Quality Research in Chinese Medicine, Macau University of Science and Technology, Taipa, Macao, Macao SAR, China; 5 Faculty of Innovation Engineering, Macau University of Science and Technology, Taipa, Macao, Macao SAR, China; 6 Department of Oncology, The Fifth Affiliated Hospital, Guangzhou Medical University, Guangzhou, Guangdong, China; 7 Baiyun District Maternal and Child Health Hospital, Guangzhou, Guangdong, China

**Keywords:** influenza virus, Liu shen wan, network pharmacology, sphingolipid metabolism, SPHK1/S1P axis

Affiliation “The Eighth School of Clinical Medicine, Guangzhou University of Chinese Medicine, Foshan, Guangdong, China.” was omitted for author Fanghao Zheng. This affiliation has now been added for author Fanghao Zheng.

Author Fanghao Zheng was erroneously assigned to affiliation “State Key Laboratory of Quality Research in Chinese Medicine, Macau University of Science and Technology, Taipa, Macau (SAR), China”. This affiliation has now been removed for author Fanghao Zheng.

The original version of this article has been updated.

