# Supplementary material for: Liu Shen Wan regulates the SPHK1/S1P axis to ameliorate influenza-induced inflammation via integrated network pharmacology and lipidomics
Source: Front Immunol. 2026 Jan 16;16:1764754. doi: 10.3389/fimmu.2025.1764754 (PMC12855086; doi:10.3389/fimmu.2025.1764754)
Supplement: Supplementary file 1 [file Table1.docx]

Supplementary Material

# 1 Supplementary Figures and Tables

## 1.1 Supplementary Figure S1


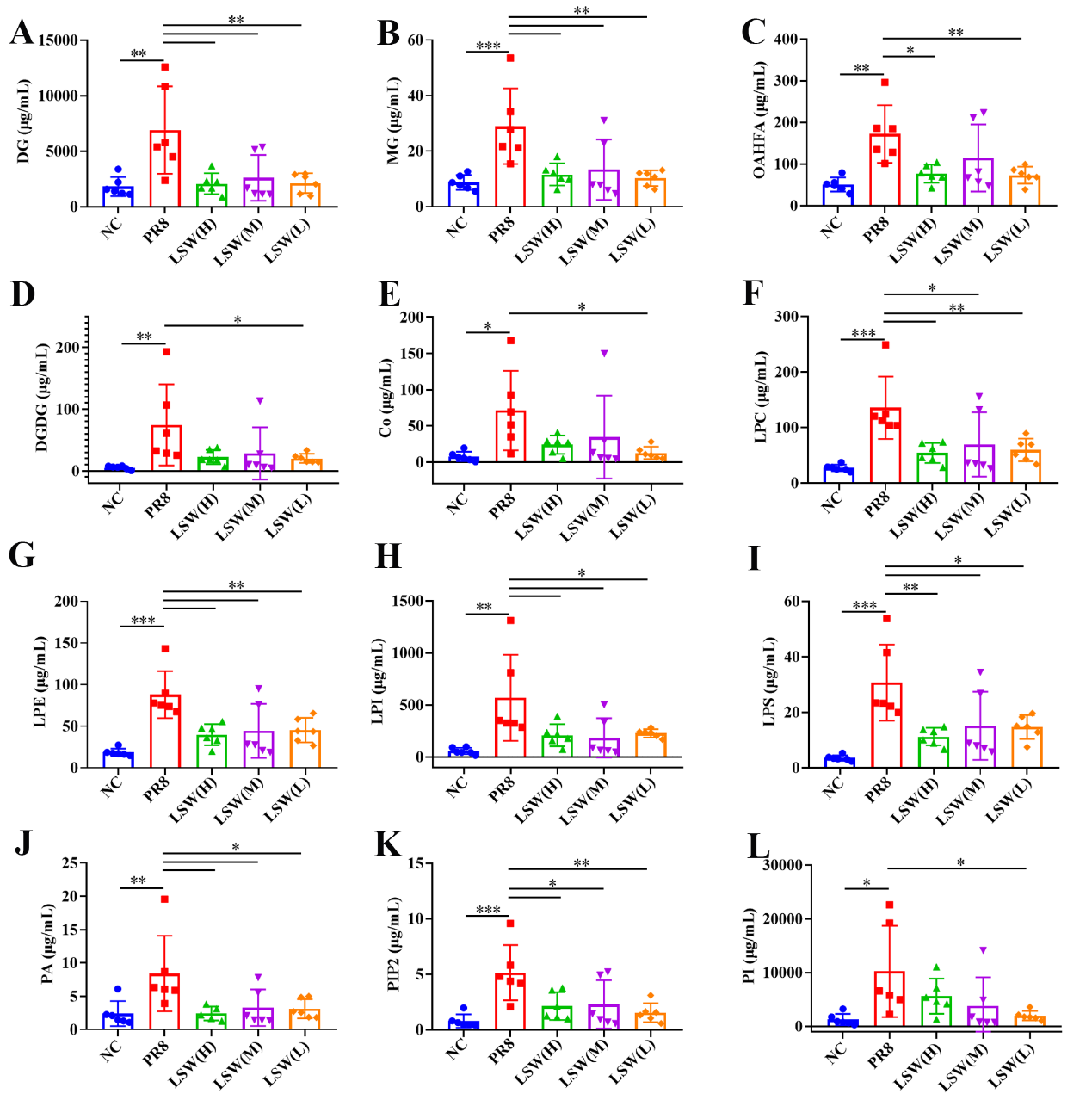


Figure S1. LSW alleviated lipid metabolism disorders in lungs during PR8 infection. (A-L) The concentration of diglyceride (DG), monoglyceride (MG), (O-acyl)-1-hydroxy fatty acid (OAHFA), digalactosyldiacylglycerol (DGDG), coenzyme (Co), lysophosphatidylcholine (LPC), lysophosphatidylethanolamine (LPE), lysophosphatidylinositol (LPI), lysophosphatidylserine (LPS), phosphatidic acid (PA), phosphatidylinositol(4,5)bisphosphate (PIP2) and phosphatidylinositol (PI) in each group.

## 1.2 Supplementary Figure S2

Figure. S2. LSW inhibited sphingolipid metabolism *in vitro*. The secretion of Cer in A549 cells during PR8 infection.

**1.3 Supplementary Table 1**

Supplementary Table 1. The sequence of SPHK1

| Gene | Sequence |
| --- | --- |
| SPHK1 | ATGTCCGCTCAAGTTCTGGGATTTTTACGCAGCTGGACTCCCCTCCCCCTGGCAGCCCCGAGGGGTCCAGCCGCCGCAGGGAATGACGCCGGTGCTCCTGCAGCCACGGCTCCGGGCGGGGAAGGCGAGCCCCACAGCCGGCCCTGCGACGCCCGCCTGGGCAGCACCGATAAGGAGCTGAAGGCAGGAGCCGCCGCCACGGGCAGCGCCCCCACAGCGCCAGGGACCCCCTGGCAGCGGGAGCCGCGGGTCGAGGTTATGGATCCAGCGGGCGGCCCCCGGGGCGTGCTCCCGCGGCCCTGCCGCGTGCTGGTGCTGCTGAACCCGCGCGGCGGCAAGGGCAAGGCCTTGCAGCTCTTCCGGAGTCACGTGCAGCCCCTTTTGGCTGAGGCTGAAATCTCCTTCACGCTGATGCTCACTGAGCGGCGGAACCACGCGCGGGAGCTGGTGCGGTCGGAGGAGCTGGGCCGCTGGGACGCTCTGGTGGTCATGTCTGGAGACGGGCTGATGCACGAGGTGGTGAACGGGCTCATGGAGCGGCCTGACTGGGAGACCGCCATCCAGAAGCCCCTGTGTAGCCTCCCAGCAGGCTCTGGCAACGCGCTGGCAGCTTCCTTGAACCATTATGCTGGCTATGAGCAGGTCACCAATGAAGACCTCCTGACCAACTGCACGCTATTGCTGTGCCGCCGGCTGCTGTCACCCATGAACCTGCTGTCTCTGCACACGGCTTCGGGGCTGCGCCTCTTCTCTGTGCTCAGCCTGGCCTGGGGCTTCATTGCTGATGTGGACCTAGAGAGTGAGAAGTATCGGCGTCTGGGGGAGATGCGCTTCACTCTGGGCACCTTCCTGCGTCTGGCAGCCCTGCGCACCTACCGCGGCCGACTGGCCTACCTCCCTGTAGGAAGAGTGGGTTCCAAGACACCTGCCTCCCCCGTTGTGGTCCAGCAGGGCCCGGTAGATGCACACCTTGTGCCACTGGAGGAGCCAGTGCCCTCTCACTGGACAGTGGTGCCCGACGAGGACTTTGTGCTAGTCCTGGCACTGCTGCACTCGCACCTGGGCAGTGAGATGTTTGCTGCACCCATGGGCCGCTGTGCAGCTGGCGTCATGCATCTGTTCTACGTGCGGGCGGGAGTGTCTCGTGCCATGCTGCTGCGCCTCTTCCTGGCCATGGAGAAGGGCAGGCATATGGAGTATGAATGCCCCTACTTGGTATATGTGCCCGTGGTCGCCTTCCGCTTGGAGCCCAAGGATGGGAAAGGTGTGTTTGCAGTGGATGGGGAATTGATGGTTAGCGAGGCCGTGCAGGGCCAGGTGCACCCAAACTACTTCTGGATGGTCAGCGGTTGCGTGGAGCCCCCGCCCAGCTGGAAGCCCCAGCAGATGCCACCGCCAGAAGAGCCCTTATAC |
